# Factors that determine patients considering medication for the disease of obesity: an IMI2 SOPHIA study

**DOI:** 10.1038/s41366-024-01524-4

**Published:** 2024-05-01

**Authors:** H. C. Craig, D. Alsaeed, H. Heneghan, W. Al-Najim, E. Al Ozairi, C. W. le Roux

**Affiliations:** 1 Diabetes Complications Research Centre, UCD Conway Institute of Biomedical and Biomolecular Research, School of Medicine, University College Dublin, Dublin, Ireland; 2 Dasman Diabetes Institute, PO Box 1180, Dasman, Kuwait; 3 Surgery, School of Medicine, University College Dublin, Dublin, Ireland

**Keywords:** Obesity, Epidemiology

## Abstract

**Objective:**

Obesity-related problems can now be managed with effective nutritional therapy, pharmacotherapy, and surgeries that achieve >10% weight loss. Assessing patient preferences, treatment choices, and factors affecting patients can improve treatment compliance and efficacy. Our aim was to identify factors affecting patient preference and subsequent choice of pharmacotherapy among those seeking treatment for obesity-related disorders.

**Methods:**

A participatory action study using purposeful sampling recruited 33 patients with obesity complications. They were referred to specialist clinics in non-alcoholic fatty liver disease, diabetes mellitus, hypertension, and chronic kidney disease. Sixteen males and seventeen females aged 18–70 years, with BMI > 35 kg/m^2^ were recruited. Before the interview, participants watched a 60-minute video explaining nutritional therapy, pharmacotherapy, and surgery in equipoise. Data were collected in semi-structured interviews; Reflective thematic analysis was used. This sub study focuses only on patients who expressed specific attitudes (positive or negative) towards pharmacotherapy.

**Results:**

Ten (30%) patients expressed a view on pharmacotherapy. Eight (24%) patients chose pharmacotherapy alone, whereas two (6%) patients chose pharmacotherapy combined with nutritional therapy. In this sub study focusing on pharmacotherapy, five themes were identified related to choosing whether or not to take medication: (1) attitudes towards pharmacotherapy, (2) attitudes toward size of obesity and its complications, (3) weighing the benefits and risks of treatment, (4) knowledge and reassurance of health professionals, and (5) costs associated with drug therapy.

**Conclusion:**

The primary concerns regarding pharmacotherapy for intentional weight loss were efficacy, side effects, lifelong dosing, pharmacokinetics, and cost. Providing access to information about all the pharmacotherapies and the benefits is likely to result in greater penetrance of treatment.

## Introduction

The World Health Organization (WHO) Obesity Report for the European Region 2022 acknowledges that no single intervention can halt the escalation of the obesity epidemic. While many member states have made good progress in improving areas of prevention and lifestyle interventions, no single member state has met their targets for stopping the increase of obesity [1]. Health care systems develop health care models evaluating two types of philosophies, such as (a) value-based care to manage costs and health care outcomes, and (b) patient-centred care which focuses on quality [2]. While there is value in both, they can only be aligned if there is a meaningful integration of patient preferences, outcomes, and perspectives incorporating them into any quality, cost and value metrics [2]. The challenge is to develop models of care which are not simply data driven but understand how patient outcomes are measured. The drive to a more activity-based funding model is one which is data driven to demonstrate improved outcomes and manage costs but must be evaluated in addition to the quality of patient centred care. Value in health care provision and the prominence of patients' right of self-determination have led to more focused integration of health services and more engagement with patients in preventing, managing and supporting them to improve their quality of life [2]. This highlights the importance of evidence-based practice. Evidence-based practice includes the best available evidence and clinical expertise, as well as taking patients’ values to provide a patient-focused, optimal treatment plan [3]. Encompassed in this is the patients’ voice, their beliefs, perceptions, and knowledge about their disease(s). The value of the patients’ voice cannot be underestimated including how their perceptions of their disease and their knowledge about the different avenues to manage/treat their obesity influence their treatment choice [4]. Patients’ voices and preferences have played an increasing role through the rise of socio-legal instruments focusing on patient autonomy in health care decision-making [5].

Pharmacotherapy to treat the disease of obesity and its complications is evolving rapidly. Moreover, awareness of their presence in the media has increased, especially in terms of effectiveness, usability, availability, and cost. The concern is that people choose medications as a quick fix. However, the use of pharmacotherapies in the treatment of obesity and obesity complications requires adherence and compliance to the treatment, and there are a number of barriers to the effective use of these medicines when patients are deciding the right treatment for them. Some of these barriers include not being convinced of the need for treatment, fear of side effects, cost, access, inadequate knowledge, patient and doctor relationship, and long-term drug regimens [6–8]. Nonetheless, in terms of intentional weight loss, patients with obesity complications are deciding what to do with limited information. Thus, patients with obesity challenges are not receiving the best patient-centred treatment for their disease.

Previously, we showed that when patients with a BMI > 35 kg/m^2^ with obesity complications are given the choice of nutritional therapy, pharmacotherapy, or surgical procedures, the factors affecting their decision in choosing obesity treatment are (1) systemic factors related to health care such as accessibility and cost, (2) autonomy including lack of knowledge and not being heard, (3) contact with formal service experienced as a lack of knowledge and support, and (4) the emotional and physical consequences of obesity where the consequences are feared to be medical [9]. This sub study aims to understand patient preferences for pharmacotherapy choices among those seeking treatment for obesity complications.

## Methodology

Participatory action research (PAR) was used to gain a deeper understanding of patients’ perspectives [10]. Our aim was to capture participants’ voices regarding pharmacotherapy treatment options. Initial data collection was analysed through thematic analysis. Interview questions were discussed with the research team and experiences from the Stratification of Phenotype in Obesity (SOPHIA) project were considered. We began by asking broad questions about patients’ experiences of their condition and asking specific questions about treatment preferences. This clarified motivations and factors affecting patients’ decisions. The interviews lasted 30–45 min to explore participants’ perspectives in depth. Ethical approval was obtained on August 6, 2021, from the Human Research Ethics Committee (HREC), University College Dublin, Ireland.

### Recruitment

Recruitment took place in specialist clinics for non-alcoholic fatty liver disease, diabetes, hypertension, and chronic kidney disease. Sixteen males and seventeen females aged 18–70 years, all with a BMI > 35 kg/m^2^ were recruited. Purposeful sampling was used to recruit 33 patients with obesity complications.

### Interviews

Data were collected in one-on-one semi-structured interviews using Zoom or telephone due to COVID-19 restrictions. Interviews were conducted by one researcher who also recruited participants at specialist clinics. Prior to the interview, participants received a link to a 60-minute video that presented three treatment options: nutritional therapy, pharmacotherapy, and surgical equivalents. Three obesity physicians were used in this presentation. Feedback was provided in a balanced manner indicating the advantages and disadvantages of each treatment. (https://www.itsnotyourfoult.ie/research).

### Data analysis

Interviews were audiotaped and transcribed verbatim. The coding framework was developed based on previous research on the topic and interview transcripts. The transcripts were anonymized and included in MAXQDA 2022 Plus software to assist with the coding of the data. Reflective thematic analysis was conducted by the first author, and a heuristic approach was used to identify and review themes and subthemes within the study [11, 12]. Data were interpreted to understand factors influencing participant selection, including motivation and the impact of obesity challenges. Additionally, content analysis was used to examine the percentage of participants who stated that they would like to choose the available obesity treatment options [13]. Based on discussions with the research team, codes and themes were refined and agreed upon using an iterative approach to foster reflexivity and dialogue, and consensus was achieved.

## Main themes

In this small study focusing on pharmacotherapy, 24% of patients chose pharmacotherapy alone, while 6% chose pharmacotherapy combined with nutritional therapy. Five main themes were identified related to participants’ opinions pertaining to pharmacotherapy (1) attitudes toward the concept of pharmacotherapy as a treatment for obesity, (2) attitudes toward obesity and its complications, (3) the benefit to risk ratio of treatment, (4) the knowledge and confidence of health professionals, and (5) the cost of pharmacotherapy. Identified themes and sub-themes shown in Table 1.

**Table 1:** Identified themes and sub-themes.

Themes	Sub-themes
The concept of pharmacotherapy as a treatment for obesity	Patients do not want to take medication for life Side effects Concerns over interactions
Perception of obesity and its complications	Society is still looking at it as a psychological failure
Benefit to risk ratio of treatment	Patient are deciding what to do and where to go with limited information
The cost of treatment	Limited access due cost being prohibitive
HCP knowledge and reassurance	Guidance of the HCP to improve compliance

### The concept of pharmacotherapy as a treatment for obesity

Assumptions about pharmacotherapy as a treatment for obesity stem from a lack of knowledge about medications’ availability and reliability. Participants reported severe concerns about medications they were already taking for other disease and the side effects of their current medications.

*‘Medication I would not know enough about. And em, I suppose I’d be in a lack of knowledge about it. I wouldn’t know enough and maybe, it’s a big jump. It’s a big leap into the unknown. You know if it didn’t work or if there were side effects, you know. Are you going to be on the wrong side of it. I just don’t know enough about it. Definitely interesting. Definitely will help someone that’s brave enough to do it, but I wouldn’t be as brave. Again, lack of knowledge. I think it’s a big jump and a big decision.’ Patient with NAFLD01*

Participants were also apprehensive about new medications interacting adversely with their current medications. Some worried about having to take the medication for life and found this frustrating. They were also concerns about regaining weight each time.

*‘I just found because of the medication and if taking the diet tablets, I’m actually on like a lot of medication like I’m on blood pressure tablets and all and I just found if I have to take medication to lose weight, you’re not going to be on for life because it’s not a long-term solution either to take it. Yeah see that’s the thing like because, like I’m already on tablets for my thyroid, blood pressure tablets, stomach tablets, and I just found fine taken another tablet will this all interact with the tablets I’m on and I’d be afraid there would be side effects down the road, like and with taking medication like I always said yeah if there was a tablet out there that would make me skinny I take it. But then when you’re looking at the pros and cons like I was like well I don’t really want to take a medication for the rest of my life, either to keep the weight off’. Patient with NAFLD02*

Those who choose pharmacotherapy can lose some weight to improve their health and hopefully stabilize their weight issues. However, some participants believed that medication treatment alone would not work without lifestyle changes.

*‘I think the medication will work when you do… you have to work with it for that to work. You’ve to eat more healthier, which I’m doing anyway, you wouldn’t think it, and be more active and I think that way it will work.’ Patient with CKD01*

*‘It would be, well, obviously weight loss would be the main goal, but to maybe retrain my brain to my appetite, you know? Right. You know, I think that’s yeah. And say, I know, I know the basics of what I need to do, you know, it’s kind of doing it is the problem, you know, so, and my sister actually is on an injection at the moment just to say, she’s diabetic. And she was put on an injection when she was diagnosed with diabetes, her GP put her and she’s getting good results out of it now in fairness. It’s slow and steady as well. It seems to be. Yeah. It’s not a huge, massive weight loss in one go, but it seems to be slow and steady with her, you know?’ Patient with CKD02*

### Perception of obesity and its complications

Participants who faced more significant challenges to their health were more inclined to view pharmacotherapy as a way to help improve their health and quality of life.

*‘Because I can’t walk. And there’s no point to me being on the list- you know the transplant list and not being able to, going in at this weight - sure they’d laugh’ Patient with CKD03*

Some participants did not choose pharmacotherapy because they agreed with the narrative that it was an easy way out of a problem, and they should be able to control it themselves.

*‘Yeah. Well, speaking from someone that’s on lots of medication, I wouldn’t go down to medication road. I’d rather do it the hard way with therapy.’ Patient with CKD04*

### Benefit to risk ratio of treatment

Participants evaluated the value of taking medication based on treatment outcomes, whether their weight loss remains stable, if their overall health improves, and whether it interacts well with existing medications they are taking.

*‘My choice would be the medication I’d be honest because I will say I, as I said to you, I’m a yoyo dieter I haven’t huge discipline when it comes to diets so I do need help and the medication would be the one I’d choose. My expectations would be sort of keeping, have a little bit of weight loss and keep the weight, get it down to a healthy BMI figure is what I’d like to try do, I don’t want to be over achieving or anything those days are gone for me, (laughs) I’m not looking for anything like that I just want to get it that, I can give myself a better lifestyle in handling the diabetes and taking control of the diabetes, that what I’d be looking for.’ Patient with NAFLD03*

### The cost of treatment

Some participants stated they were seeking a more direct solution and reported cost as a consideration to accessing any medication.

*‘And then I obviously when I went to the chemist to get it the chemist thought I had diabetes, that kind of way. And they told me, “Just say nothing, just see what they say, just hand in the script and see what way.” And that was fine, the first month was free. And then came the next month they rang me and says, “Oh, I’m ringing, you’re not entitled to this.” And I was paying €150 a month for me medication as it was, so I couldn’t afford to pay another 100 a month. So, we stopped that. And then COVID took over and the clinic went. But that would be the medication side, it’d be more so the cost that I couldn’t afford it because I’ve no medical card, you know what I mean?’ Patient with CKD05*

### HCP knowledge and reassurance

Participants reported that the knowledge and reassurance provided by their health care professional (HCP) had a significant impact on treatment choices. Clear explanations and good communication of each treatment plan are essential for the patient to make informed decisions. Participants valued the HCPs views, particularly regarding if the treatment with pharmacotherapy is needed, if they confirmed it would not affect existing conditions negatively, and if there was support and follow up.

*‘I know Novo Nordisk have now taken Ozempic and got a stronger dosing for obesity. That’s something I’d like to talk to the doctors about as I go forward.’ Patient with CKD06*

## Discussion

People with obesity complications identified weight loss outcomes and effects on their obesity related complications and quality of life as major influencing factors in their choices. Their decisions were influenced by their beliefs and attitudes towards their HCP and the information, communication strategies, and recommendations provided about the need for this treatment. Participants explained that they received some information about medication availability through the media, but this did not influence their choice. Most information pertaining to pharmacotherapy came from their HCP, which highlights the value for improved health literacy. Their choices were influenced mainly by the perception of their health conditions, their health beliefs, and their relationship with their doctors which was deemed of paramount importance when choosing pharmacotherapy. Participants had concerns around side effects, availability of support, follow up, and taking the medication for life. Several participants addressed the issue of medication adherence. Beena et al. (2011) distinguished between medication adherence and compliance. Medication compliance means that the patient complies with the physician’s authority, while medication adherence refers to a collaboration between physician and patient in an effort to improve patient health [7]. Bissell emphasized how communication between HCPs and patients should be viewed as a possible way to combine the expertise of both to agree on mutual goals [10]. This concept of flexibility is where an understanding of patient and provider beliefs and preferences is important when deciding interventions [11]. Knowing and understanding what the patient wants to treat obesity in this way can prevent conflict [12].

A number of studies have taken different approaches to analysing patient with other chronic diseases and their preferences from systematic literature reviews to qualitative interviews. They all identified cost, side effects, knowledge, and the health benefit which is consistent with the themes found in this study. For example, Gomez-Peralta et al. (2021) conducted a quantitative study on patient preferences for pharmacological diabetes treatment conducting a questionnaire with 238 participants. They found the most important aspects for patients was health outcomes, adverse events, treatment characteristics, and treatment costs. They concluded that people with diabetes prefer treatments that reduced blood pressure and their HbA1c level [13]. Muhlbacher et al. (2014) conducted a literature review on patient preferences in treatment of diabetes mellitus: Thirteen studies were included in the analysis and they found that blood sugar control, side effects and long term complications as well as the mode of administration were the most patient relevant outcomes [14]. Xu X et al. (2022) conducted a systematic literature review on patient preferences, expectations and value for the management and treatment of hypertension. They identified that the side effects, cost and convenience were important factors for patients [15]. They also identified that patient’s preferred shared decision making on treatment options. In a qualitative study conducted by Neus Pages-Puigdemont et al. (2016) on patients’ perspective of medication adherence in chronic conditions, 36 participants were interviewed and they found that the participants health beliefs and perceptions of disease control impact adherence in chronic patients [8]. They also highlighted the importance of the patient-HCP relations and recommended further research to focus on shared decision making and more health education [8].

Patients’ voices and preferences have played an increasing role [5] and this has led to a stronger focus on shared decision making highlighting the importance of clear, concise communication, information and a collaborative relationship between the HCP and the patient [8]. This highlights the need to improve health literacy for patients, improve knowledge for HCPs, and increase support for those wishing this therapeutic approach. Further research is recommended on how people can access information around new pharmacotherapy options as well as the value of increasing access to this treatment pathway.

The limitations of the study include that the primary focus was on understanding the choices of patients between nutritional therapies, pharmacotherapies, and surgical therapies. This sub study to understand decision making of patients regarding pharmacotherapy was not the primary objective, albeit that the rich data allows novel insights. The sample size is relatively small, but in qualitative research, the sample size is not determined by a power calculation, but rather by the ‘theoretical sufficiency’ [16] of the data. This sufficiency is determined by the quality of the data collected—their richness, depth, diversity and complexity, what can be glossed as data or sampling adequacy – rather than the quantity of data collected [16–18]. Thus, based on the saturation which occurred in the emerging themes from this work, recruiting more patients would not have yielded additional themes. The patients that were recruited were not representative of the wider global population, but qualitative research is not extrapolatable because the data is only relevant to the patients studied within the qualitative study.

The strengths of the study include that the patients who were interviewed were unselected with regards to their enthusiasm for any obesity treatment. The initial launch for treatments which effectively addresses complications of obesity such as type 2 diabetes, including semaglutide and tirzepatide, has exceeded all expectations [5, 19–21]. This resulted in a shortage of medications and a prevailing view that most patients living with obesity want to be treated with medication. However, the estimates are that in late 2023, only 8% of patients with obesity were on pharmacotherapy. Thus, it is not possible to estimate at present if medications were available without any supply restrictions and how many people living with obesity would be receiving pharmacotherapy. As such, there may be a “ceiling” beyond which pharmacotherapy cannot easily grow. For example, if bariatric surgery is made freely available to the public with very few limitations for access, such as in Belgium or Luxembourg, fewer than 3% of the eligible population will select to have bariatric surgery. It is therefore important to understand what are the factors which would prevent a large majority of people living with obesity to consider pharmacotherapy for the treatment of the disease of obesity.

## Conclusion

In conclusion, the main patient concerns with medication for the disease of obesity are efficacy, side effects, the requirement for lifelong treatment, and the cost. The main advantage of the medication is the perceived utility of the treatment of the disease of obesity and how this may reduce existing and future complications of obesity. This highlights the need to improve patient health literacy, improve knowledge for health care professions, and increase support for those who wish to benefit from this treatment modality.

The importance of the study: Health outcomes can be improved by understanding patient preferences and incorporating them into obesity treatment. This study reports factors affecting decisions regarding pharmacotherapy for obesity include perceptions of pharmacotherapy, perceptions of obesity and its complications, weighing the benefits and risks of treatment, and knowledge and reassurance by HCPs.

## Data Availability

The datasets generated during and/or analysed during the current study are available from the corresponding author on reasonable request.
